# 2-Chloro-*N*,*N*-diphenyl­acetamide

**DOI:** 10.1107/S1600536809024052

**Published:** 2009-06-27

**Authors:** Shuai Shao, Jie Sun

**Affiliations:** a College of Food Science and Light Industry, Nanjing University of Technology, Xinmofan Road No. 5 Nanjing, Nanjing 210009, People’s Republic of China

## Abstract

In the title compound, C_14_H_12_ClNO, the central acetamide plane forms dihedral angles of 76.0 (2) and 64.0 (2)° with the phenyl rings and the phenyl rings form a dihedral angle of 71.8 (2)° with each other.

## Related literature

The title compound is an important inter­mediate in the synthesis of *N*-phenyl-indolin-2-one, which can be further transformed to l-aryl-3-(amino­alkyl­idene)oxindoles, a new class of ‘GABAergic’ agents (Shindikar *et al.*, 2006 ▶; Sarges *et al.*, 1989 ▶) using a new variant of the Friedel–Crafts cyclization (Hennessy & Buchwald, 2003 ▶; Trost & Frederiksen, 2005 ▶; Trost & Yong, 2006 ▶).
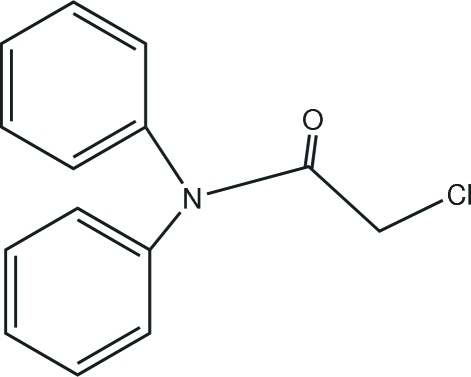


## Experimental

### 

#### Crystal data

C_14_H_12_ClNO
*M* *_r_* = 245.70
Orthorhombic, 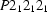

*a* = 6.4350 (13) Å
*b* = 12.799 (3) Å
*c* = 14.944 (3) Å
*V* = 1230.8 (5) Å^3^
*Z* = 4
Mo *K*α radiation
μ = 0.29 mm^−1^
*T* = 293 K
0.30 × 0.20 × 0.10 mm

#### Data collection

Enraf–Nonius CAD-4 diffractometer
Absorption correction: ψ scan (North *et al.*, 1968 ▶) *T*_min_ = 0.917, *T*_max_ = 0.971
2519 measured reflections
2231 independent reflections
1842 reflections with *I* > 2σ(*I*)
*R*_int_ = 0.064
3 standard reflections every 200 reflections intensity decay: 1%

#### Refinement

*R*[*F* ^2^ > 2σ(*F* ^2^)] = 0.045
*wR*(*F* ^2^) = 0.112
*S* = 1.00
2231 reflections
154 parameters
H-atom parameters constrained
Δρ_max_ = 0.18 e Å^−3^
Δρ_min_ = −0.21 e Å^−3^
Absolute structure: Flack (1983 ▶), 912 Friedel pairs
Flack parameter: −0.14 (9)

### 

Data collection: *CAD-4 Software* (Enraf–Nonius, 1989 ▶); cell refinement: *CAD-4 Software*; data reduction: *XCAD4* (Harms & Wocadlo, 1995 ▶); program(s) used to solve structure: *SHELXS97* (Sheldrick, 2008 ▶); program(s) used to refine structure: *SHELXL97* (Sheldrick, 2008 ▶); molecular graphics: *PLATON* (Spek, 2009 ▶); software used to prepare material for publication: *SHELXL97* and *PLATON*.

## Supplementary Material

Crystal structure: contains datablocks global, I. DOI: 10.1107/S1600536809024052/ya2097sup1.cif

Structure factors: contains datablocks I. DOI: 10.1107/S1600536809024052/ya2097Isup2.hkl

Additional supplementary materials:  crystallographic information; 3D view; checkCIF report

## Figures and Tables

**Fig. 1.**
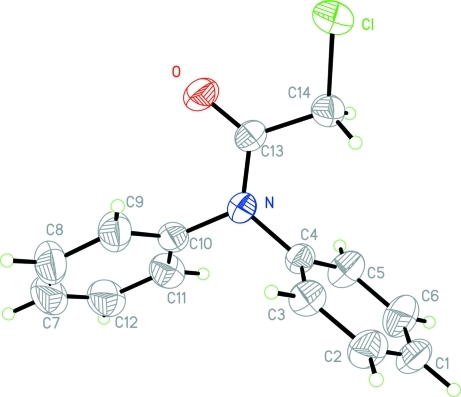
Molecular structure of the title compound; thermal displacement ellipsoids are drawn at the 50% probability level. H atoms are shown as small circles of arbitrary radius.

## supplementary crystallographic information

### Comment

The title compound is an important intermediate in the synthesis of *N*-phenyl-indolin-2-one, which can be further transformed to l-aryl-3-(aminoalkylidene)oxindoles, a new class of "GABAergic" agents (Shindikar *et al.*, 2006; Sarges *et al.*, 1989) using the new variant of the Friedel-Crafts cyclization (Hennessy & Buchwald, 2003; Trost & Frederiksen, 2005; Trost & Yong, 2006).

In the molecule of the title compound (Fig 1), dihedral angles formed by the central plane C14/C13/N/O with phenyl rings C1—C6 and C7—C12 are equal to 104.0 (2)° and 116.0 (2)° respectively; phenyl rings form dihedral angle 108.2 (2)° with each other.

### Experimental

The title compound was prepared by refluxing for 2 hrs of the mixture of diphenylamine (1.69 g, 0.01 mol) and chloroacetyl chloride (1.13 g, 0.01 mol) in 50 ml of toluene. 150 ml of water was then added to the reaction mixture causing precipitation of the product, which was filtered, washed with water, dried and and recrystallized from ethanol (yield 97%). Crystals suitable for X-ray analysis were obtained by slow evaporation of a chloroform solution (yield 96%, m.p.413 K).

### Refinement

The H atoms were positioned geometrically (C—H 0.97 and 0.93 Å for methylene and aromatic H, respectively), and included in the refinement in the riding motion approximation with *U*_iso_(H) = 1.2*U*_eq_ of the carrying atom.

### Crystal data

**Table:**

C_14_H_12_ClNO	*D*_x_ = 1.326 Mg m^−^^3^
*M**_r_* = 245.70	Melting point: 393 K
Orthorhombic, *P*2_1_2_1_2_1_	Mo *K*α radiation, λ = 0.71073 Å
Hall symbol: P 2ac 2ab	Cell parameters from 25 reflections
*a* = 6.4350 (13) Å	θ = 9.0–13.0°
*b* = 12.799 (3) Å	µ = 0.29 mm^−^^1^
*c* = 14.944 (3) Å	*T* = 293 K
*V* = 1230.8 (5) Å^3^	Block, colorless
*Z* = 4	0.30 × 0.20 × 0.10 mm
*F*(000) = 512	

### Data collection

**Table:**

Enraf–Nonius CAD-4 diffractometer	1842 reflections with *I* > 2σ(*I*)
Radiation source: fine-focus sealed tube	*R*_int_ = 0.064
graphite	θ_max_ = 25.3°, θ_min_ = 2.1°
ω/2θ scans	*h* = −7→0
Absorption correction: ψ scan (North *et al.*, 1968)	*k* = −15→15
*T*_min_ = 0.917, *T*_max_ = 0.971	*l* = −17→0
2519 measured reflections	3 standard reflections every 200 reflections
2231 independent reflections	intensity decay: 1%

### Refinement

**Table:**

Refinement on *F*^2^	Secondary atom site location: difference Fourier map
Least-squares matrix: full	Hydrogen site location: inferred from neighbouring sites
*R*[*F*^2^ > 2σ(*F*^2^)] = 0.045	H-atom parameters constrained
*wR*(*F*^2^) = 0.112	*w* = 1/[σ^2^(*F*_o_^2^) + (0.065*P*)^2^] where *P* = (*F*_o_^2^ + 2*F*_c_^2^)/3
*S* = 1.00	(Δ/σ)_max_ < 0.001
2231 reflections	Δρ_max_ = 0.18 e Å^−^^3^
154 parameters	Δρ_min_ = −0.21 e Å^−^^3^
0 restraints	Absolute structure: Flack (1983), 912 Friedel pairs
Primary atom site location: structure-invariant direct methods	Flack parameter: −0.14 (9)

### Special details

**Table:**

Geometry. All e.s.d.'s (except the e.s.d. in the dihedral angle between two l.s. planes) are estimated using the full covariance matrix. The cell e.s.d.'s are taken into account individually in the estimation of e.s.d.'s in distances, angles and torsion angles; correlations between e.s.d.'s in cell parameters are only used when they are defined by crystal symmetry. An approximate (isotropic) treatment of cell e.s.d.'s is used for estimating e.s.d.'s involving l.s. planes.
Refinement. Refinement of *F*^2^ against ALL reflections. The weighted *R*-factor *wR* and goodness of fit *S* are based on *F*^2^, conventional *R*-factors *R* are based on *F*, with *F* set to zero for negative *F*^2^. The threshold expression of *F*^2^ > σ(*F*^2^) is used only for calculating *R*-factors(gt) *etc*. and is not relevant to the choice of reflections for refinement. *R*-factors based on *F*^2^ are statistically about twice as large as those based on *F*, and *R*- factors based on ALL data will be even larger.

### Fractional atomic coordinates and isotropic or equivalent isotropic displacement parameters (Å^2^)

**Table:**

	*x*	*y*	*z*	*U*_iso_*/*U*_eq_	
Cl	0.97222 (13)	0.79305 (6)	0.11836 (6)	0.0621 (3)	
O	1.0050 (3)	0.63002 (15)	−0.01779 (12)	0.0490 (5)	
N	1.2460 (3)	0.52988 (16)	0.05137 (14)	0.0354 (5)	
C1	1.6477 (5)	0.4811 (2)	0.2686 (2)	0.0543 (8)	
H1A	1.7359	0.4711	0.3172	0.065*	
C2	1.4544 (5)	0.4359 (2)	0.26845 (19)	0.0514 (8)	
H2A	1.4111	0.3956	0.3167	0.062*	
C3	1.3243 (5)	0.4509 (2)	0.19570 (17)	0.0410 (7)	
H3A	1.1943	0.4193	0.1943	0.049*	
C4	1.3879 (4)	0.51270 (19)	0.12568 (16)	0.0343 (6)	
C5	1.5827 (5)	0.5572 (2)	0.1258 (2)	0.0485 (7)	
H5A	1.6263	0.5978	0.0777	0.058*	
C6	1.7120 (5)	0.5409 (3)	0.1976 (2)	0.0596 (9)	
H6A	1.8439	0.5706	0.1981	0.072*	
C7	1.1939 (8)	0.2816 (3)	−0.1312 (2)	0.0726 (12)	
H7A	1.1834	0.2270	−0.1719	0.087*	
C8	1.0262 (7)	0.3081 (3)	−0.0781 (3)	0.0695 (11)	
H8A	0.9034	0.2702	−0.0825	0.083*	
C9	1.0392 (5)	0.3908 (2)	−0.0181 (2)	0.0527 (8)	
H9A	0.9261	0.4090	0.0174	0.063*	
C10	1.2238 (5)	0.4453 (2)	−0.01240 (17)	0.0381 (7)	
C11	1.3920 (5)	0.4182 (2)	−0.06415 (18)	0.0500 (8)	
H11A	1.5165	0.4546	−0.0594	0.060*	
C12	1.3728 (7)	0.3355 (3)	−0.1236 (2)	0.0626 (9)	
H12A	1.4858	0.3170	−0.1590	0.075*	
C13	1.1327 (4)	0.6186 (2)	0.04201 (16)	0.0341 (6)	
C14	1.1818 (4)	0.7040 (2)	0.10923 (18)	0.0410 (6)	
H14A	1.3058	0.7412	0.0905	0.049*	
H14B	1.2092	0.6729	0.1672	0.049*	

### Atomic displacement parameters (Å^2^)

**Table:**

	*U*^11^	*U*^22^	*U*^33^	*U*^12^	*U*^13^	*U*^23^
Cl	0.0602 (5)	0.0518 (5)	0.0743 (6)	0.0188 (4)	−0.0070 (4)	−0.0153 (4)
O	0.0505 (12)	0.0557 (12)	0.0409 (10)	0.0128 (10)	−0.0121 (10)	−0.0018 (9)
N	0.0378 (12)	0.0378 (12)	0.0306 (11)	0.0016 (11)	−0.0061 (10)	−0.0002 (10)
C1	0.055 (2)	0.063 (2)	0.0450 (17)	0.0191 (17)	−0.0191 (17)	−0.0039 (16)
C2	0.066 (2)	0.0570 (18)	0.0315 (15)	0.0058 (16)	−0.0011 (15)	0.0079 (13)
C3	0.0404 (16)	0.0448 (15)	0.0377 (15)	−0.0018 (13)	0.0028 (13)	0.0058 (13)
C4	0.0360 (14)	0.0374 (13)	0.0295 (13)	0.0037 (11)	−0.0034 (12)	−0.0008 (11)
C5	0.0403 (16)	0.0593 (18)	0.0459 (17)	−0.0076 (13)	−0.0032 (14)	0.0137 (15)
C6	0.0380 (18)	0.078 (2)	0.063 (2)	−0.0050 (16)	−0.0138 (17)	−0.0025 (19)
C7	0.122 (4)	0.0484 (19)	0.047 (2)	0.006 (2)	−0.023 (2)	−0.0115 (16)
C8	0.086 (3)	0.0488 (19)	0.073 (2)	−0.020 (2)	−0.029 (2)	0.0012 (17)
C9	0.057 (2)	0.0487 (17)	0.0525 (17)	−0.0111 (16)	−0.0092 (16)	0.0001 (15)
C10	0.0508 (17)	0.0327 (14)	0.0307 (14)	0.0016 (13)	−0.0058 (13)	0.0035 (11)
C11	0.060 (2)	0.0472 (17)	0.0429 (16)	0.0021 (15)	0.0058 (16)	−0.0007 (14)
C12	0.086 (3)	0.0569 (19)	0.0447 (18)	0.012 (2)	0.000 (2)	−0.0085 (16)
C13	0.0329 (14)	0.0406 (14)	0.0288 (13)	0.0003 (12)	0.0009 (12)	0.0045 (11)
C14	0.0385 (14)	0.0403 (15)	0.0443 (15)	0.0026 (12)	0.0001 (13)	−0.0028 (13)

### Geometric parameters (Å, °)

**Table:**

Cl—C14	1.771 (3)	C6—H6A	0.9300
O—C13	1.223 (3)	C7—C12	1.347 (6)
N—C13	1.357 (3)	C7—C8	1.382 (6)
N—C10	1.449 (3)	C7—H7A	0.9300
N—C4	1.454 (3)	C8—C9	1.390 (5)
C1—C2	1.372 (5)	C8—H8A	0.9300
C1—C6	1.372 (5)	C9—C10	1.380 (4)
C1—H1A	0.9300	C9—H9A	0.9300
C2—C3	1.386 (4)	C10—C11	1.375 (4)
C2—H2A	0.9300	C11—C12	1.386 (4)
C3—C4	1.374 (4)	C11—H11A	0.9300
C3—H3A	0.9300	C12—H12A	0.9300
C4—C5	1.377 (4)	C13—C14	1.518 (4)
C5—C6	1.374 (4)	C14—H14A	0.9700
C5—H5A	0.9300	C14—H14B	0.9700
			
C13—N—C10	120.3 (2)	C7—C8—C9	120.7 (3)
C13—N—C4	122.9 (2)	C7—C8—H8A	119.7
C10—N—C4	116.8 (2)	C9—C8—H8A	119.7
C2—C1—C6	120.5 (3)	C10—C9—C8	118.5 (3)
C2—C1—H1A	119.8	C10—C9—H9A	120.8
C6—C1—H1A	119.8	C8—C9—H9A	120.8
C1—C2—C3	119.4 (3)	C11—C10—C9	121.0 (3)
C1—C2—H2A	120.3	C11—C10—N	118.7 (3)
C3—C2—H2A	120.3	C9—C10—N	120.2 (3)
C4—C3—C2	119.8 (3)	C10—C11—C12	118.9 (3)
C4—C3—H3A	120.1	C10—C11—H11A	120.6
C2—C3—H3A	120.1	C12—C11—H11A	120.6
C3—C4—C5	120.6 (3)	C7—C12—C11	121.4 (4)
C3—C4—N	118.8 (2)	C7—C12—H12A	119.3
C5—C4—N	120.7 (2)	C11—C12—H12A	119.3
C6—C5—C4	119.3 (3)	O—C13—N	122.4 (2)
C6—C5—H5A	120.3	O—C13—C14	122.5 (2)
C4—C5—H5A	120.3	N—C13—C14	115.0 (2)
C1—C6—C5	120.4 (3)	C13—C14—Cl	110.84 (19)
C1—C6—H6A	119.8	C13—C14—H14A	109.5
C5—C6—H6A	119.8	Cl—C14—H14A	109.5
C12—C7—C8	119.6 (3)	C13—C14—H14B	109.5
C12—C7—H7A	120.2	Cl—C14—H14B	109.5
C8—C7—H7A	120.2	H14A—C14—H14B	108.1
			
C6—C1—C2—C3	0.2 (5)	C8—C9—C10—N	−178.0 (3)
C1—C2—C3—C4	−1.6 (4)	C13—N—C10—C11	116.2 (3)
C2—C3—C4—C5	2.1 (4)	C4—N—C10—C11	−65.9 (3)
C2—C3—C4—N	−178.1 (2)	C13—N—C10—C9	−66.3 (3)
C13—N—C4—C3	100.9 (3)	C4—N—C10—C9	111.6 (3)
C10—N—C4—C3	−77.0 (3)	C9—C10—C11—C12	0.9 (4)
C13—N—C4—C5	−79.4 (3)	N—C10—C11—C12	178.4 (2)
C10—N—C4—C5	102.8 (3)	C8—C7—C12—C11	−0.7 (5)
C3—C4—C5—C6	−1.3 (4)	C10—C11—C12—C7	−0.2 (5)
N—C4—C5—C6	179.0 (3)	C10—N—C13—O	2.1 (4)
C2—C1—C6—C5	0.7 (5)	C4—N—C13—O	−175.7 (2)
C4—C5—C6—C1	−0.1 (5)	C10—N—C13—C14	−175.8 (2)
C12—C7—C8—C9	1.1 (5)	C4—N—C13—C14	6.4 (4)
C7—C8—C9—C10	−0.4 (5)	O—C13—C14—Cl	23.3 (3)
C8—C9—C10—C11	−0.6 (4)	N—C13—C14—Cl	−158.8 (2)
